# SINs and SOMs: neural microcircuits for size tuning in the zebrafish and mouse visual pathway

**DOI:** 10.3389/fncir.2013.00089

**Published:** 2013-05-10

**Authors:** Alison J. Barker, Herwig Baier

**Affiliations:** ^1^Department Genes-Circuits-Behavior, Max Planck Institute of NeurobiologyMartinsried, Germany; ^2^Neuroscience Graduate Program, University of California San FranciscSan Francisco, CA, USA

**Keywords:** optic tectum, visual cortex, zebrafish (*Danio rerio*), size discrimination, inhibitory interneurons

## Abstract

In many animals, a fast and reliable circuit for discriminating between predator-sized objects and edible (prey-sized) objects is necessary for survival. How are receptive fields (RFs) in visual brain areas organized to extract information about size? Recent studies from the zebrafish optic tectum and the mouse visual cortex suggest *de novo* shaping of RFs by subtypes of inhibitory neurons. Del Bene et al. (2010) describe a population of GABAergic neurons in the zebrafish optic tectum (superficial interneurons, SINs) that are necessary for size filtering during prey capture. Adesnik et al. (2012) describe a somatostatin-expressing interneuron population (SOMs) that confers surround suppression on layer II/III pyramidal cells in mouse V1. Strikingly both the SINs and the SOMs, display size-dependent response properties. Increasing visual stimulus size increases excitatory input to these neurons. Dampening SIN or SOM activity alters tuning of neighboring circuits such that they lose preference for small objects. Both results provide exciting evidence for mechanisms of size filtering in visual circuits. Here we review the roles of the SINs and the SOMs and speculate on the similarity of such spatial filters across species.

## THE SINs

The pursuit and capture of small prey (e.g., *paramecia*) by the zebrafish larva require that information about the size and motion of the prey object be continually tracked. Larvae with laser ablations of the optic tectum are unable to perform this behavior (Gahtan et al., 2005), and several studies have identified neurons in the tectum with preferential size tuning to prey-sized objects (Sajovic and Levinthal, 1982a,b; Niell and Smith, 2005; Muto et al., 2013). Del Bene et al. (2010) searched for the locus of small object tuning in the tectum. Retinal ganglion cell axons enter the tectum largely in its superficial layers (Robles et al., 2013). Visual information is then transmitted through synaptic circuitry to the deeper layers of the tectal neuropil, from where it is carried on to the motor centers of the midbrain and hindbrain. The resident neurons in the deep layers of the tectum are the periventricular neurons (PVNs). They comprise two main classes: periventricular interneurons (PVINs) make only local connections in the tectum, whereas the periventricular projection neurons (PVPNs) receive inputs from PVIN axons in the deeper layers and send efferent axons to premotor and motor areas (Nevin et al., 2010). Only some classes of PVINs send dendrites to the superficial, retinorecipient layers.

**FIGURE 1:**
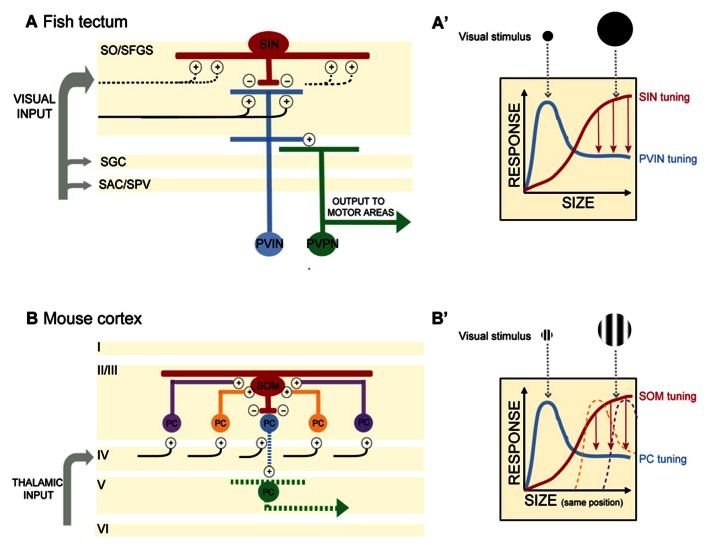
**(A)** In the optic tectum of the zebrafish larva, superficial interneurons (SINs) are preferentially tuned to large objects in the visual field. Periventricular neurons (PVNs) with dendrites stratifying in the deep neuropil are preferentially tuned to small objects. PVINs denote periventricular interneurons, PVPNs, periventricular projection neurons. Retinal inputs distribute among four main layers of the tectum (SO, stratum opticum; SFGS, stratum fibrosum et griseum superficiale; SGC, stratum griseum centrale; SAC/SPV, stratum album centrale/stratum periventriculare). SIN cell bodies are located in the SO and extend dendritic and axonal arbors throughout the SFGS. SINs may receive excitatory synaptic input directly from retinal ganglion cell axons or from PVINs or both. Plus and negative signs denote excitatory and inhibitory connections, respectively. Blue and red indicate excitatory and inhibitory interneurons, respectively. Projection neurons are colored in green. Black lines depict retinal ganglion cell axons. Dashed lines indicate predicted, but not yet demonstrated synaptic connections. **(A′)** Schematic of SIN filtering in the optic tectum. As the size of the visual stimulus increases, SINs become activated and provide inhibitory input to sharpen the tuning of PVIN receptive fields. Size-tuning curves for SINs and PVINs are depicted by red and blue curves, respectively. Red arrows denote inhibition acting to sharpen tuning. **(B) **In the mouse visual cortex, somatostatin-expressing interneurons (SOMs) are preferentially tuned to large objects in the visual field and display no surround suppression. SOMs are positioned in layer II/III and receive lateral excitatory inputs from pyramidal cells (PCs) in layer II/III. SOMs provide inhibition to neighboring layer II/III PCs, resulting in surround suppression and PC preferential tuning to smaller objects. Cortical layers I to VI are denoted. Plus and negative signs denote excitatory and inhibitory connections, respectively. Red denotes inhibitory interneurons. Excitatory PCs in layer II/III are colored in blue, orange, and purple (corresponding to their relative distance from the SOM). The central PC (shown in blue) receives surround suppression from the SOM. PCs in layer V are colored in green. For simplicity in this schematic, only connections pertaining to size filtering circuitry are shown. Projections of layer II/III PCs are not shown. Black lines depict input from the thalamus. Dashed lines indicate predicted, but not yet demonstrated synaptic connections. **(B′)** Schematic of SOM filtering in the visual cortex. Here the visual stimulus is a drifting grating (within a circular aperture) of increasing size. Size tuning of the central PC shown in (**B**; blue) is depicted here with a blue size-tuning curve. As the stimulus size increases beyond the size of the blue PC’s receptive field, the receptive fields of neighboring PCs are activated. The large visual stimulus occupies the same position in the visual field as the small stimulus, but increases in diameter thus encompassing multiple PC receptive fields (depicted here by the orange and purple size-tuning curves). These PCs provide excitatory drive to a SOM. SOM activation in turn provides surround suppression to the blue PC. The size-tuning curve of the SOM is depicted in red. Red arrows denote inhibition acting to sharpen tuning.

By selectively expressing genetically encoded calcium indicators (GCaMP1.6 and 3) in retinal ganglion cell axons, Del Bene and colleagues (2010) found that retinal afferents displayed uniform activity regardless of stimulus size. On the other hand, dendrites of PVNs (presumably a mix of PVINs and PVPNs) stratifying within the deep layers of the tectal neuropil were preferentially tuned to small moving bars, whereas many PVIN dendrites in the superficial neuropil were responsive to both full-field visual stimuli (here a full screen flash) and small moving bars. The characteristic tuning to small moving objects of less than 10° was observed in many single PVNs and across populations of PVNs (**Figures 1A,A′**). Dampening GABAergic tone through local application of bicuculline increased Ca^2+^ responses to large objects, suggesting that GABAergic control normally sieves information by size as it trickles down to the deep layers. How is this achieved?

Del Bene et al. (2010) identified a population of GABAergic interneurons, the superficial interneurons (SINs), positioned in the superficial tectum. Using a transgenic Gal4 line that allowed them to target these cells – they showed that SINs are preferentially tuned to wide-field visual stimuli (**Figures 1A,A′**). When presented with a moving bar of increasing width, SINs expressing GCaMP displayed increasing Ca^+2^ responses as the size of the bar increased. Selectively ablating the SINs by photoactivation of KillerRed protein resulted in a loss of small object preference in the deep tectal layers. Importantly, the KillerRed experiments demonstrate that size tuning in the tectum is not inherited solely (if at all) from the retina. Rather intratectal circuits substantially contribute to size tuning. In addition, synaptically silencing SINs through genetically targeted expression of tetanus toxin decreased performance of larvae in a prey capture assay, providing a link between the size tuning for small objects in the deep neuropil and size-discrimination behavior. The optomotor response (OMR) requires the detection of large-field motion and is not dependent on an intact tectum (Roeser and Baier, 2003). As expected silencing of the SINs had no measurable effect on the OMR (Del Bene et al., 2010).

Already in 1982, Sajovic and Levinthal observed that tectal neurons can be optimally tuned to objects much smaller than their receptive fields (RFs), and smaller even than the RFs of retinal ganglion cells providing visual input. Sajovic and Levinthal (1982a,b) suggested that inhibition was responsible for this size tuning, but the exact nature of the inhibitory mechanism remained elusive. A piece of this puzzle has been resolved in the identification of the SINs, but it is likely that there are additional sources of inhibition acting in the tectum. Conversely, it is possible that the SINs have additional functions in filtering incoming visual inputs.

## THE SOMs

A key feature of visual cortical neurons is their selective tuning to both the size and orientation of objects in the visual field (Angelucci and Bressloff, 2006). Adesnik et al. (2012) investigated the contribution of surround suppression to size tuning in the mouse visual cortex (V1). In awake behaving mice, drifting gratings were presented in a circular aperture of increasing size to determine the preferred stimulus size for pyramidal cells (PCs) and two populations of inhibitory neurons in layer II/III in V1. PC size tuning was determined through extracellular recordings of single units, while loose patch recordings were employed to determine size tuning in parvalbumin-expressing neurons (PVs) and somatostatin-expressing neurons (SOMs). PCs displayed preferred tuning to relatively small apertures (around 22°), while PVs and SOMs preferred tuning was for larger apertures (**Figures 1B,B′**). Both PCs and PVs exhibited surround suppression (as the stimulus size increased outside the RF of the neuron, firing rate decreased). Significantly, in addition to having larger RFs than PCs and PVs, the SOMs exhibited no surround suppression.

How are inputs to SOMs structured to generate large RFs lacking surround suppression? Adesnik et al. (2012) determined that SOMs sum excitation across visual space through lateral excitation from PCs in layer II/III. While recording from SOMs and directly activating layer IV PCs expressing channelrhodopsin (ChR2) little excitation in the SOMs was observed. In contrast, ChR2-mediated activation of layer II/III PCs resulted in large increases in excitatory drive to SOMs. By simultaneously recording from PCs in layer II/III, the authors were able to make comparisons between SOM activity and PC activity while stimulating each layer. Unlike the lateral excitatory drive onto SOMs, PVs appeared to receive the majority of their excitatory input from layer IV PCs.

In electrophysiology experiments performed while expressing halorhodopsin (NpHR) in SOMs and ChR2 in layer II/III PCs, the authors confirmed that layer II/III PC activation resulted in increased excitatory input and spiking in SOMs with the opposite effect on non-ChR2 expressing PCs – increased inhibitory postsynaptic currents (IPSCs) and decreased spiking. When SOMs were silenced with NpHR activation during these dual recordings from SOMs and neighboring PCs, SOM spiking was reduced and inhibition of PCs was lost (measured by decreased IPSCs). These experiments suggested that SOMs are responsible for the PC inhibition observed during the presentation of a large visual stimulus (**Figure 1B′**). The results of Adesnik et al. (2012) are similar to the findings of Del Bene et al. (2010) in demonstrating that (1) there is an anatomically identifiable microcircuit for size filtering and (2) size filtering is not solely transmitted by input from earlier stages of visual processing, but can be computed directly in visual brain regions.

## SOMe SINsible OPEN QUESTIONS

Some details of SIN and SOM circuitry remain obscure. How are inputs to SOMs and SINs organized? Adesnik et al. (2012) demonstrate direct synaptic connections between SOMs and neighboring PCs in layer II/III, yet the lateral extent of SOM inhibition across layer II/III is unknown. For example, how many PCs send input to a single SOM? Similarly, how many PCs receive inhibition from a single SOM? It is also unclear how SOM inhibition shapes the output of the system. Are RFs in layer V neurons (the site of projection neurons to other brain regions) also changed when SOMs are silenced? SINs have a direct effect on size-dependent behavioral responses in the zebrafish. How might eliminating SOMs affect visually mediated behaviors in the mouse?

The local circuitry of the SINs in the fish tectum is even less well understood. To what cells are the SINs synaptically connected? Taking a note from the SOMs, one might predict that SINs receive input from PVNs mapping adjacent areas of visual space and provide feedback inhibition onto PVNs to modulate PVN firing for a maximum response to small objects. More likely, SINs receive direct retinal input and provide feedforward inhibition to PVNs, restricting their size tuning (see **Figure 1A**). Detailed electrophysiology experiments as performed by Adesnik et al. (2012), are needed in this system.

## LOOKING FORWARD

One interesting question is how other inhibitory populations contribute to shape size tuning. Might multiple filters for small-sized objects exist? Or filters for large or medium-sized objects? Additional interneuron populations have been described in the visual cortex and tectum (e.g., Kerlin et al., 2010; Robles et al., 2011). It will also be worthwhile to explore if SIN and SOM mechanisms for size filtering are employed by other visual brain areas. In the mouse, retinal input is not exclusively channeled to the cortex. Substantial retinal input arrives in the superior colliculus (SC). Evidence of surround suppression has been reported in the superficial layers of mouse SC, where the majority of cells are optimally tuned to small objects (6°–10°) and display decreased responsiveness at larger stimulus sizes (Wang et al., 2010). Further characterization of GABAergic populations in the mouse SC will be necessary to determine if SIN/SOM-like mechanisms are at work in this visual brain region.

One behavioral implication of size-filtering circuitry is the ability to recognize edible objects during prey capture. This behavior is impaired when SIN function is perturbed (Del Bene et al., 2010). Predator avoidance, the recognition and avoidance of large objects, is equally important for an organism’s survival. Avoidance behavior in many species can be elicited through the presentation of a looming stimulus, a two-dimensional representation of an object on a collision course. For looming objects it is not just the size of the object that is important rather its rate of expansion, taking into account the size and speed of the approaching object (Fotowat and Gabbiani, 2011). Loom-sensitive neurons have been detected in the mouse retina (Münch et al., 2009). Might the SINs or SOMs be part of a loom-detecting circuit? Additional studies to probe speed and size tuning of SINs and SOMs may provide valuable insights into their potential role in avoidance behaviors.

Despite differences in methodology and model organisms, a unifying principle emerges from these studies: size tuning relies on local inhibition to reshape RFs and filter out wide-field visual inputs. While the tectum is homologous to the mammalian SC it is striking that mechanisms for size filtering are similar between species and across visual brain areas. It is therefore tempting to extend these findings to other sensory systems where the role of local inhibition may act to refine and reshape RFs. This may be necessary to ensure the fidelity of synaptic transmission, increase single-to-noise ratios or allow for greater flexibility in extracting relevant information from raw sensory input. The results in mouse visual cortex demonstrate how local inhibition can shape RFs in visual brain regions. The zebrafish tectum findings provide a clear link between inhibition-modulated size tuning in visual brain regions and behavior that relies on size discrimination. This work provides one final lesson – that these small vertebrates have a lot to tell us about neural circuits and perception.

## Conflict of Interest Statement

The authors declare that the research was conducted in the absence of any commercial or financial relationships that could be construed as a potential conflict of interest.

## References

[B1] Adesnik H. Bruns W. Taniguchi H. Huang Z. J. Scanziani M. (2012). A neural circuit for spatial summation in visual cortex. *Nature* 490 226–231 23060193 10.1038/nature11526 PMC3621107

[B2] Angelucci A. Bressloff P. C. (2006). Contribution of feedforward, lateral and feedback connections to the classical receptive field center and extra-classical receptive field surround of primate V1 neurons. *Prog. Brain Res.* 154 93–120 17010705 10.1016/S0079-6123(06)54005-1

[B3] Del Bene F. Wyart C. Robles E. Tran A. Looger L. Scott E. K. (2010). Filtering of visual information in the tectum by an identified neural circuit. *Science* 330 669–673 21030657 10.1126/science.1192949 PMC3243732

[B4] Fotowat H. Gabbiani F. (2011). Collision detection as a model for sensory-motor integration. *Annu. Rev. Neurosci.* 34 1–19 21391815 10.1146/annurev-neuro-061010-113632 PMC13285341

[B5] Gahtan E. Tanger P. Baier H. (2005). Visual prey capture in larval zebrafish is controlled by identified reticulospinal neurons downstream of the tectum. *J. Neurosci.* 25 9294–9303 16207889 10.1523/JNEUROSCI.2678-05.2005 PMC6725764

[B6] Kerlin A. M. Andermann M. L. Berezovskii V. K. Reid R. C. (2010). Broadly tuned response properties of diverse inhibitory neuron subtypes in mouse visual cortex. *Neuron* 67 858–871 20826316 10.1016/j.neuron.2010.08.002 PMC3327881

[B7] Münch T. A. da Silveira R. A. Siegert S. Viney T. J. Awatramani G. B. Roska B. (2009). Approach sensitivity in the retina processed by a multifunctional neural circuit. *Nat. Neurosci*. 12 1308–1316 19734895 10.1038/nn.2389

[B8] Muto A. Ohkura M. Abe G. Nakai J. Kawakami K. (2013). Real-time visualization of neuronal activity during perception. *Curr. Biol*. 23 307–311 23375894 10.1016/j.cub.2012.12.040

[B9] Nevin L. M. Robles E. Baier H. Scott E. K. (2010). Focusing on optic tectum circuitry through the lens of genetics. *BMC Biol.* 8:126 10.1186/1741-7007-8-126 PMC2949621 20920150

[B10] Niell C. M. Smith S. J. (2005). Functional imaging reveals rapid development of visual response properties in the zebrafish tectum. *Neuron* 45 941–951 15797554 10.1016/j.neuron.2005.01.047

[B11] Robles E. Smith S. J. Baier H. (2011). Characterization of genetically targeted neuron types in the zebrafish optic tectum. *Front. Neural Circuits* 5:1 10.3389/fncir.2011.00001 PMC3046383 21390291

[B12] Robles E. Filosa A. Baier H. (2013). Precise lamination of retinal axons generates multiple parallel input pathways in the tectum. *J. Neurosci.* 33 5027–5039 23486973 10.1523/JNEUROSCI.4990-12.2013 PMC3641828

[B13] Roeser T. Baier H. (2003). Visuomotor behaviors in larval zebrafish after GFP-guided laser ablation of the optic tectum. *J. Neurosci.* 23 3726–3734 12736343 10.1523/JNEUROSCI.23-09-03726.2003 PMC6742205

[B14] Sajovic P. Levinthal C. (1982a). Visual cells of zebrafish optic tectum: mapping with small spots. *Neuroscience* 7 2407–2426 7177381 10.1016/0306-4522(82)90204-4

[B15] Sajovic P. Levinthal C. (1982b). Visual response properties of zebrafish tectal cells. *Neuroscience* 7 2427–2440 7177382 10.1016/0306-4522(82)90205-6

[B16] Wang L. Sarnaik R. Rangarajan K. Liu X. Cang J. (2010). Visual receptive field properties of neurons in the superficial superior colliculus of the mouse. *J. Neurosci*. 30 16573–16584 21147997 10.1523/JNEUROSCI.3305-10.2010 PMC3073584

